# Bugs scaring bugs: enemy‐risk effects in biological control systems

**DOI:** 10.1111/ele.13601

**Published:** 2020-09-09

**Authors:** Michael Culshaw‐Maurer, Andrew Sih, Jay A. Rosenheim

**Affiliations:** ^1^ Department of Entomology and Nematology University of California Davis CA 95616 USA; ^2^ Department of Evolution and Ecology University of California Davis CA 95616 USA; ^3^ Department of Environmental Science and Policy University of California Davis CA 95616 USA

**Keywords:** Agricultural ecology, behavioural ecology, biological control, enemy‐risk effects, natural enemies, non‐consumptive effects, pest management, predation risk, predator–prey ecology, trophic cascades

## Abstract

Enemy‐risk effects, often referred to as non‐consumptive effects (NCEs), are an important feature of predator–prey ecology, but their significance has had little impact on the conceptual underpinning or practice of biological control. We provide an overview of enemy‐risk effects in predator–prey interactions, discuss ways in which risk effects may impact biocontrol programs and suggest avenues for further integration of natural enemy ecology and integrated pest management. Enemy‐risk effects can have important influences on different stages of biological control programs, including natural enemy selection, efficacy testing and quantification of non‐target impacts. Enemy‐risk effects can also shape the interactions of biological control with other pest management practices. Biocontrol systems also provide community ecologists with some of the richest examples of behaviourally mediated trophic cascades and demonstrations of how enemy‐risk effects play out among species with no shared evolutionary history, important topics for invasion biology and conservation. We conclude that the longstanding use of ecological theory by biocontrol practitioners should be expanded to incorporate enemy‐risk effects, and that community ecologists will find many opportunities to study enemy‐risk effects in biocontrol settings.

## Graphical abstract

We provide a synthetic review of enemy‐risk effects in biological control of arthropod pests. We provide a conceptual overview of enemy‐risk effects, review the current body of literature documenting risk effects in biocontrol systems and describe in detail ways in which risk effects may impact the implementation of biocontrol programs.

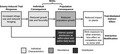

## INTRODUCTION

Biological control (or biocontrol) is the use of an organism to reduce or prevent the unwanted impact of another organism, typically through an exploitative interaction (Eilenberg *et al*., 2001). While competitive relationships are sometimes utilised (Tyndale‐Biscoe and Vogt, 1996), most biological control agents, including predators, parasitoids, pathogens and herbivores, are consumers of pest organisms (Heimpel and Mills, 2017). Perhaps the best‐known form of biological control is ‘classical’ or importation biological control, where a natural enemy is imported from a region other than the target area, often from the native home range of the pest. Today, this involves a rigorous process of enemy selection, efficacy testing and non‐target testing (Bigler *et al*., 2006), since history is filled with examples of exotic enemies wreaking havoc on naïve, native communities (Simberloff and Stiling, 1996). Inundative and inoculative releases of natural enemies, collectively referred to as ‘augmentative control’, involve the release of large numbers of enemies, either to bolster existing populations or to provide a short pulse of control without long‐term establishment. In contrast, conservation biological control is the attempt to increase the effectiveness of already‐present enemies. Methods include the provision of alternative resources for biocontrol agents (e.g. extrafloral or floral nectar, pollen), changes in landscape complexity and the preservation of natural areas beneficial to enemies (Bianchi *et al*., 2006; Tscharntke *et al*., 2007, 2016). Altogether, these various methods of biological control provide significant ecosystem services in both natural and agricultural ecosystems (Losey and Vaughan, 2006; Zhang and Swinton, 2012; Naranjo *et al*., 2015).

Biological control and predator–prey/parasitoid‐host (‘natural enemy’) ecology have a long relationship (Hassell and Varley, 1969; McMurtry *et al*., 1970; Murdoch *et al*., 1985). Early theory in natural enemy ecology was heavily influenced by examples of classical biological control, and broader natural enemy ecology has served to inform biocontrol practice. However, biological control has lagged behind natural enemy ecology by not recognising the impact and importance of enemy‐risk effects, often referred to as non‐consumptive effects (NCEs), fear effects, risk effects, non‐lethal effects or trait‐mediated effects. Biocontrol typically focuses on direct lethal effects of enemies on pests, whether through consumption or parasitism (which we refer to as consumptive effects or CEs) or through infection. However, natural enemy ecology has long recognised the importance of enemy‐risk effects (Abrams *et al*., 1996; Werner and Anholt, 1996; Schmitz, 1998; Werner and Peacor, 2003). Enemies induce behavioural, physiological, morphological or life‐history changes in their prey that can lead to significant changes in individual fitness, population dynamics and community dynamics. Meta‐analyses and reviews have noted that even when natural enemies kill relatively few prey or hosts, they can have major impacts via enemy‐risk effects (Preisser *et al*., 2005; Peckarsky *et al*., 2008; Preisser and Bolnick, 2008; Schmitz *et al*., 2008; Sih *et al*., 2010; Buchanan *et al*., 2017). While numerous studies have demonstrated major enemy‐risk effects in many biological control systems, this knowledge has not been implemented in standard thinking about biocontrol. Several ways that enemy‐risk effects connect to biocontrol include understanding: (1) the dynamics of trophic cascades where natural enemies have positive impacts on plants not only by killing pests (CEs), but also by altering pest traits; (2) the role of risk effects in governing interactions in biocontrol systems with multiple enemies, intraguild predation (IGP) and bottom‐up effects; (3) the impacts of enemy‐induced pest dispersal on the spatiotemporal ecology of biocontrol; and (4) how effects of natural enemies differ on coevolved versus naïve prey, as is common for target versus non‐target prey respectively. Insights about enemy‐risk effects can thus help to better guide agent selection, non‐target testing, integrated pest management (IPM) programs and other biocontrol practices. Conversely, biocontrol systems are ideal for the general study of enemy‐risk effects, offering opportunities to study risk at multiple scales, across multiple trophic levels, with varying levels of co‐evolution, and in systems amenable to experimental manipulation.

We provide a systematic overview of insights gained from integrating enemy‐risk effects into the ecology of biocontrol, focusing on management of arthropod pests. We begin with a conceptual overview of current literature on enemy‐risk effects, including work outside of biocontrol systems, then review studies of enemy‐risk effects in biocontrol and finish by demonstrating and discussing in some detail how a conceptual knowledge of risk effects can inform and improve pest management and biocontrol programs (see Box 1 for a well‐studied example).

Box 1 **Enemy‐risk effects and the biological control of the red imported fire ant**

The red imported fire ant, *Solenopsis invicta*, was inadvertently introduced from South America into the port city of Mobile, Alabama in the 1930s. Expanding its range across much of the southern United States, it achieved exceptionally high densities (5–10 times greater than in their native range), displacing native ants, damaging agricultural production and creating a sting hazard for anyone active outdoors (Porter and Gilbert, 2004; Oi *et al*., 2015). After a massive and controversial insecticide‐based eradication effort failed, attention turned to classical biological control. Studies in the native range of the ants revealed over 20 species of parasitoid flies in the genus *Pseudacteon* (family Phoridae), most of which appeared to be host specific and thus to be potentially acceptable in terms of low risk of non‐target impacts. *Pseudacteon* spp. parasitoids lay eggs in adult worker ants, the resulting parasitoid larvae completing their development in the heads of their host ants, which fall off as the larvae develop (hence their common name: decapitating flies).

Early investigations of *Pseudacteon* spp. in the native ranges of the fire ants concluded, however, that they were poor candidates for effective biological control, because they achieved very low rates of parasitism (Jouvenaz *et al*. 1981). Extensive year‐long sampling across multiple sites confirmed that parasitism was indeed rare, with only 0.24% of workers parasitised on average (Calcaterra *et al*., 2008). Retrospective analyses of the extensive literature on the introductions of parasitoids as classical biological control agents by Hawkins *et al*. (1993) and Hawkins and Cornell (1994) suggested that a threshold for success exists: parasitoids that fail to achieve maximum parasitism rates of >32% in their native ranges, or >33‐36% in their introduced ranges, have been unable to produce economically acceptable levels of pest suppression. Because the entire *Pseudacteon* spp. complex exerted a maximum of only 2.81% parasitism in the native range (Calcaterra *et al*., 2008), the suggestion that these flies would be of ‘dubious value’ for biological control (Jouvenaz *et al*. 1981) was not hard to understand.

However, as argued by Feener and Brown (1992) and Porter and Gilbert (2004), a reliance on parasitism rates alone might lead us to grossly underestimate the potential value of *Pseudacteon* spp. parasitoids as control agents. Earlier studies had shown that phorid parasitoids attacking a different ant, while also generating little parasitism, elicited dramatic anti‐predator defences. Ants responded to the presence of flies by fleeing back to the nest or by sheltering from fly attacks in the leaf litter, causing the ants to lose their status as competitive dominants in their interactions with other ants (Feener, 1981). Subsequent studies of *S. invicta* revealed a similar pattern: in response to a fly’s arrival, workers retreated underground, took cover below sticks or pebbles, or adopted stereotypic defensive postures with their sting‐bearing gasters raised (Orr *et al*., 1995; Porter *et al*., 1995). This eliminated their ability to recruit foragers to food sources, with other ants immediately exploiting the now‐available resources. Just a single parasitoid could arrest the foraging activity of hundreds of fire ant workers (Porter *et al*., 1995). Thus, *S. invicta* display dramatic and costly anti‐predator defences, and the non‐consumptive effects of phorid flies on fire ants may allow native ants to compete effectively with these invaders.

Thus, recognition of the potential importance of enemy‐risk effects of *Pseudacteon* spp. motivated the decision to import these species as classical biological control agents. Six species have been introduced to the United States to date, with different species attacking different subsets of worker ants, based on ant size, time of activity or foraging location (at the nest or at foraging trails; reviewed by Oi *et al*., 2015). Importantly, host‐range testing included assessments not only of parasitism of non‐targets, but also the attraction to worker ants and expression of the hovering attacks that elicit defensive responses (Porter and Gilbert, 2004). Whether the enemy‐risk effects will prove to be sufficient to control *S. invicta* in its invasive range remains, however, an open question, as *Pseudacteon* spp. continue to build their populations and expand their ranges while monitoring continues (Chen and Fadamiro, 2018; Oi *et al*., 2019).

## ENEMY‐RISK EFFECTS: A BRIEF CONCEPTUAL OVERVIEW

Many organisms exhibit responses to natural enemies (predators and parasitoids; we frequently use ‘predator/prey’ as a catchall that includes parasitoid/host relationships), including within‐generation changes in behaviour (e.g reduced activity, increased refuge use, increased group size; Lima, 1998), physiology (Hawlena and Schmitz, 2010; Clinchy *et al*., 2013), morphology (Bourdeau and Johansson, 2012; Hulthén *et al*., 2014) and life history (Miner *et al*., 2005; LaManna and Martin, 2016; Relyea *et al*., 2018). These responses typically have costs in terms of reduced feeding and growth rates and, ultimately, reduced fitness (Kerfoot and Sih, 1987; Stamps, 2007; Orrock *et al*., 2013) and population growth rates (Creel and Christianson, 2008). Because these responses often involve niche shifts (e.g. in prey diets or habitat use), they also affect prey interactions with other species (Werner and Peacor, 2003). For example, anti‐predator responses can alter competition among prey (Werner and Anholt, 1996), increase exposure to other predators (Sih *et al*., 1998; Fouzai *et al*., 2019) or to diseases (Duffy *et al*., 2011; Shang *et al*., 2019) and alter impacts on their own resources (Schmitz *et al*., 2004). Notably, if prey exhibit strong, effective anti‐enemy responses, predators might actually kill few prey (i.e. have weak consumptive effects, CEs) but instead have large impacts on prey fitness and prey interactions with the rest of their community (Preisser *et al*., 2005). These three levels of effect (individual response, impacts on fitness/populations and community effects) are best defined as ***enemy‐induced trait responses***, ***non‐consumptive effects*** and ***trait‐mediated indirect effects*** (Peacor *et al*., 2020). Box 2 discusses this terminology in greater detail.

Box 2 **Categorising enemy‐risk effects**

**Figure 1:**
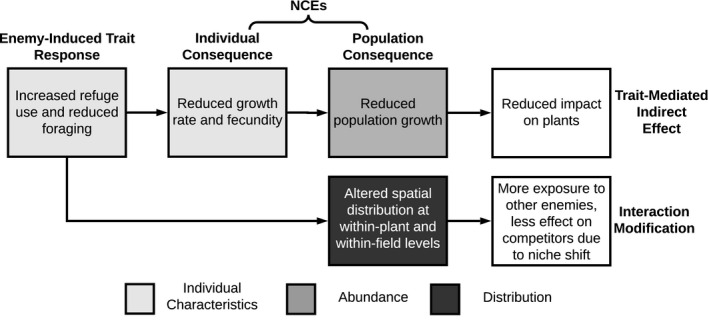
Demonstration of a particular enemy‐risk effect fitting in to the broader framework we describe in Box 2. An enemy‐risk effect is described by both the stage, beginning with individual response and ending with community effects, as well as by the effects on the abundance, distribution and characteristics of a pest population.

The term NCE is frequently used to describe processes at three levels: the enemy‐induced trait response (e.g. increased refuge use), the consequences for the individual prey/host (e.g. reduced growth rate) or the consequences at the prey/host population level (e.g. increased emigration). Referring to all three levels as NCEs reduces the important distinctions between them, we advocate for a more explicit framework (Fig. 1), and clearer terminology (also see Peacor *et al*., 2020). We will use the terms ***enemy‐risk effect*** to refer to the overall process, ***enemy‐induced trait response*** to refer to the mechanism of response, ***NCE*** to refer to fitness/population consequences and ***trait‐mediated indirect effect*** to refer to effects cascading to trophic levels below the prey/host. A complementary way of conceptualising enemy‐risk effects is to take a more phenomenological approach, focusing on the aspects of a pest population: its *per capita* impact, abundance and distribution (box shading in Fig. 1).

**Behavioural shifts** are a commonly studied trait responses in arthropods, and are generally the most rapid and reversible. Examples include changes in time spent feeding (Thaler and Griffin, 2008; Jandricic *et al*., 2016; Ingerslew and Finke, 2017), food source (Schmitz *et al*., 1997), microhabitat and refuge use (Lucas *et al*., 2000; Lawson‐Balagbo *et al*., 2007; Penfold *et al*., 2017), oviposition rate (Deas and Hunter, 2013; Hermann and Thaler, 2018), oviposition site selection (Angelon and Petranka, 2002; Vonesh and Blaustein, 2010; Silberbush and Blaustein, 2011), short‐distance escape (Tamaki *et al*., 1970; Nelson, 2007; Fill *et al*., 2012) and dispersal (Höller *et al*., 1994; Henry *et al*., 2010; Welch and Harwood, 2014; Otsuki and Yano, 2014b).

**Physiological shifts** can be direct responses to risk, but they are often the consequences of behavioural shifts. For example, a reduction in individual growth rate (physiological) is often a result of reduced foraging effort (behavioural). This can make physiological shifts difficult to categorise within the framework shown in Fig. 1. Examples include changes in growth rate (Kaplan *et al*., 2014), development time (Bellamy and Alto, 2018) and assimilation efficiency (Thaler *et al*., 2014).

**Morphological shifts** are generally slower to appear and more difficult to reverse than behavioural or even physiological shifts. They have been less described in terrestrial arthropods, but thoroughly studied in systems such as *Daphnia pulex*, where predator cues trigger production of carapace protrusions that decrease vulnerability to predation (Havel and Dodson, 1984; Tollrian, 1995; Rabus and Laforsch, 2011). **Life‐history shifts** frequently occur over a long timescale and are irreversible for an individual prey/host. They include changes in timing of reproduction or metamorphosis (Ims, 1990; Benard, 2004; Relyea, 2007), quality and quantity of offspring produced (Map pes *et al*., 1997) and production of winged morphs (Sloggett and Weisser, 2002; Kunert and Weisser, 2003).

Trait responses carry costs for individuals, and we can categorise **NCEs** based on these costs. These costs are ultimately tied to individual fitness, including reduced fecundity (Map pes *et al*., 1997) and reduced survival (Walzer and Schausberger, 2009).

Both responses and consequences at the individual level can cascade to affect the entire prey/host population. Finally, community‐level impacts include both **trait‐mediated indirect effects**, wherein an NCE reduces the prey population such that they have a smaller effect on a lower trophic level, and **interaction modifications**, wherein a trait response causes an existing interaction with another species to change. As seen in Fig. 1, these community effects can occur via different pathways that may not be captured equally in all studies.

Experiments to evaluate the relative strength of CEs and NCEs typically contrast the total effect of actual predators (CE + NCEs) with the effect of constrained predators (e.g. predators caged or artificially manipulated to prevent use of mouthparts) or predator cues (NCEs only) on prey. A meta‐analysis of these experiments found that the importance of NCEs was highly variable, but on average roughly the same magnitude as CEs (Preisser *et al*., 2005). For biocontrol, trait‐mediated indirect effects cascading to the plant may be even more relevant. Enemies frequently have very strong positive effects on plants due to trait shifts by herbivores, even when CEs are relatively small (Schmitz *et al*., 2004; Creel and Christianson, 2008).

Behavioural ecology theory and experiments suggest that prey typically exhibit stronger trait responses when perceived risk is higher and when the marginal costs of response are lower (Lima, 1998). When perceived risk reflects actual risk, predators that are more dangerous in the absence of prey defences can induce such strong anti‐predator responses that they kill fewer prey (but cause stronger NCEs) than less dangerous predators. Thus, predation rate is often not a good measure of predation risk, and therefore not always a good indicator of total effect on prey (CEs + NCEs). Perceived risk, however, is not always proportional to actual risk. Perceived risk depends on not just the type of predator and its attack success, but also on the type and strength of predator cues or prey alarm cues (Kats and Dill, 1998; Stankowich and Blumstein, 2005; Ferrari *et al*., 2010), on the habitat *per se* (Verdolin, 2006; Thaker *et al*., 2011) and on prey sensory/cognitive capacities (Kats and Dill, 1998; Ferrari *et al*., 2010; Bedoya‐Perez *et al*., 2019). Predators that are not very dangerous, but difficult to locate and assess (e.g. ambush predators) can induce strong anti‐predator responses and thus strong NCEs (Sih, 1992; Preisser *et al*., 2007). Prey may even respond to an organism that is incapable of killing them if the cues are sufficiently close to those of a dangerous enemy (Fill *et al*., 2012). Box 3 discusses how prey perceive risk in more detail and implications for enemy‐risk effects and biocontrol.

Box 3 **Prey perception of risk**

A large literature in behavioural and sensory ecology has examined prey perception of danger based on cues that provide information on the levels of enemy risk (Weissburg *et al*., 2014; Ehlman *et al*., 2019). Arthropods perceive risk using chemical (both airborne and via direct contact; Dicke and Grostal, 2001; Sitvarin and Rypstra, 2012; Hermann and Thaler, 2014), visual (Gonçalves‐Souza *et al*., 2008), vibratory (Castellanos and Barbosa, 2006), auditory (Skals, 2005) and tactile cues (Castellanos *et al*., 2011; Okada and Akamine, 2012). Organisms often use multiple cue modalities, which can vary depending on prey perceptual ability and the types of enemies.

A primary source of risk cues is the enemy itself, whether directly as sounds, vibrations, chemical cues or visual presence, or indirectly as chemical footsteps, faeces, molts and silk. Organisms can also respond to indicators of risk before they actually detect enemies; for example by responding to ‘alarm cues’ associated with other prey being attacked, injured or killed (Schoeppner and Relyea, 2005; Vandermoten *et al*., 2012). Alarm cues can induce a range of responses and can even be shared across species (Agarwala *et al*., 2003; Goodale and Nieh, 2012). Another cue may be habitat or microhabitat type. If certain habitat types are associated with enemy risk, then risk avoidance may drive habitat selection, regardless of direct cues from enemies or even conspecifics (Lucas *et al*., 2000).

Cues can vary widely in spatiotemporal extent, affecting different numbers of prey over varying timescales. For example, because chemical cues can spread widely and remain detectable for long periods, they can cause risk effects to persist long after enemies have left an area (Wilson and Leather, 2012; Ninkovic *et al*., 2013). Theory suggests that because the cost of under‐responding to risk (i.e. getting killed) is often much greater than the cost of over‐responding (e.g. hiding unnecessarily and losing feeding opportunities), when cues provide imprecise information about the presence (versus absence) of predators, this uncertainty can induce strong enemy‐risk effects even when predators are only occasionally present (Sih, 1992). This may be true for many prey facing the risk of attack by ambush predators. In contrast, seeing or coming into physical contact with an enemy is usually a more definitive risk indicator.

The links between cue generation, detection and anti‐enemy response are complex, involving multiple steps and interactions. Environmental context can strongly affect both the strength and detection of a cue (e.g. wind may disperse a chemical cue) and the perception of risk upon detection (e.g. perceived risk may be lower if a refuge is nearby). Response to risk can be highly state dependent; a starving organism may be more likely to accept higher risk to avoid starvation, and a larger, faster individual may assess risk differently than a smaller, more vulnerable organism. In some cases, it can take a combination of multiple cues to trigger a response (Gish *et al*., 2011). Recent theoretical work has suggested that cues indicating risk should be integrated with other cues indicating safety to shape responses (Trimmer *et al*., 2017; Ehlman *et al*., 2019), and supporting evidence has emerged from recent studies with desert isopods (Zaguri and Hawlena, 2019).

A key insight from signal detection theory is that all cues are imperfect indicators. Cues can vary in strength; a chemical cue can be diluted or concentrated, a visual cue can be obscured by other objects and an auditory cue can be disrupted by ambient sounds. On top of variance in cue strength, the specificity of cue modalities can vary. The visual cue of a looming shape could come from a dangerous enemy or a harmless passing organism, the chemical and tactile cue of a parasitoid could come from a species that parasitises the pest or another, closely related parasitoid that does not (Fill *et al*., 2012), and cues that elicit stress and reduce population growth can come from activity of commensal organisms (Jensen and Toft, 2020). The reliability of cues may change with the introduction of novel organisms (Ehlman *et al*., 2019) or through habituation to the cue. The consistent application of synthetic alarm pheromone may cause decreased sensitivity of aphids to the cue, but this insensitivity may in turn increase CEs by coccinellid predators (de Vos *et al*., 2010). Finally, synthetic predator kairomones can increase mosquito mortality synergistically with *Bacillus thuringiensis* applications, even when completely decoupled from real predators (Op de Beeck *et al*., 2016; Delnat *et al*., 2020). Biocontrol practices might benefit from deeper understanding of pest perception of cues associated with enemy risk.

Marginal costs of enemy‐induced trait responses are higher (and prey exhibit weaker trait responses) if prey are energy stressed (hungry), resources or mates are abundant but more accessible only if prey show little anti‐predator response, or if prey have high reproductive value (more to lose; Houston *et al*., 1999; Clark, 1994). For herbivores, the strength of the enemy‐risk effect depends on, among other things, plant abundance and quality, herbivore condition and life‐history stage (McArthur *et al*., 2012; Stephan *et al*., 2017).

The role of enemy‐risk effects in community dynamics becomes more complex when we consider multiple enemies and IGP, common occurrences in biocontrol systems. With multiple agents of mortality, enemy‐risk effects can often blend into CEs where a trait response to an enemy (e.g. a shift in microhabitat use) increases mortality from another enemy (Sih *et al*., 1998), environmental stressors (Schmitz *et al*., 1997) or even pesticides (Janssens and Stoks, 2013). With IGP, predators are also potentially prey, and thus also exhibit enemy‐induced trait responses and NCEs. The mix of CEs and NCEs involving multiple species then influences community outcomes including biocontrol efficacy. We discuss this in more detail in a later section.

Many of these predictions about enemy‐risk effects assume that prey exhibit adaptive responses to enemies that they have coevolved with. Prey lacking evolutionary (or developmental) history with enemies (or specific enemies) often exhibit much weaker anti‐enemy responses and thus suffer heavy mortality (strong CEs) when novel enemies appear (e.g. island prey or prey in fishless ponds; Cox and Lima, 2006; Carthey and Blumstein, 2018). This depends on the cue or functional similarity of new enemies to the prey’s familiar enemies (Sih *et al*., 2010; Carthey and Banks, 2014; Saul and Jeschke, 2015). Given that biocontrol often involves introducing enemies that have a co‐evolutionary history with the target pest, but not with non‐target organisms, the effect of evolutionary history on CEs versus NCEs is clearly a salient issue that we discuss in more detail below.

## LITERATURE REVIEW OF BIOLOGICAL CONTROL ENEMY‐RISK EFFECT STUDIES

We carried out a systematic review of empirical studies on enemy‐risk effects in biocontrol systems using combinations of the search terms “biological control”, “biocontrol”, and “pest” with the terms “non‐consumptive”, “nonconsumptive”, “non‐lethal”, “nonlethal”, “sub‐lethal”, “sublethal”, “risk effect*”, “anti‐predator”, or “anti‐predator.” Studies were included if they were on arthropod pests, investigated some stage of the enemy‐risk effect pathway depicted in Fig. 1, and demonstrated some relevance to pest control. Our review of the literature yields several takeaway messages: (1) enemy‐risk effects are prevalent in arthropod pest systems, (2) enemy‐induced trait shifts can interact with other aspects of agroecosystems, such as plant defences, trap crops and plant pathogen transmission, (3) risk effects produced by predators have been studied more extensively than those produced by parasitoids, (4) the importance of enemy‐risk effects on non‐target species has received little attention and (5) few studies have examined the consequences of enemy‐risk effects for plant damage in the field.

We organised papers in Table 1 according to the ‘level’ of study, ranging from documentation of enemy‐induced trait responses to explicit measure of NCEs on pest control and trait‐mediated indirect effects on crops (see Supporting Information for expanded table format). This categorisation is not meant to rank the quality or usefulness of studies, but rather to demonstrate where research has been focused and where room for growth remains. Fifty‐four per cent of studies (32 of 59) aimed to assess the strength of pest responses, which is a critical step in the inclusion of enemy‐risk effects in the design and implementation of biocontrol programs. Many of these studies incorporated other aspects relevant to pest management, such as variation in spatial scale (Lee *et al*., 2014), ability to transmit plant pathogens (Tholt *et al*., 2018), interactions with trap cropping (Lee *et al*., 2011) and plant defence (Thaler *et al*., 2014). Of the 27 remaining studies, about half documented demographic consequences for pests, and half documented the levels of pest damage. Four studies measured changes in plant damage in the field (Griffin and Thaler, 2006; Thaler and Griffin, 2008; Steffan and Snyder, 2010; Hermann and Thaler, 2018). Only two studies measured the risk effects of enemies on non‐target species (Walzer and Schausberger, 2009; Fill *et al*., 2012); these effects are likely overlooked in many evaluations of host range, as we discuss in the following section.

**TABLE 1:** Table of biocontrol enemy‐risk effect studies, organised according to the level of study

‘Highest’ level of study	Other aspects	Citation
Behavioural/Physiological/Morphological Response (32)	None (8)	Angelon and Petranka, (2002), Silberbush *et al*. (2010), Silberbush and Blaustein, (2011), Warburg *et al*. (2011), Thaler *et al*. (2012), Fischhoff *et al*. (2018), Dupuy and Ramirez, (2019), La‐Spina *et al*. (2019)
	Variation among agents (8)	Pallini *et al*. (1999), Wuellner *et al*. (2002), Ramirez *et al*. (2010), Hoki *et al*. (2014), Otsuki and Yano, (2014b), Dias *et al*. (2016), Jacobsen *et al*. (2016), Staats *et al*. (2016)
	Variation among pests (1)	Wilson and Leather, (2012)
	Variation among agents and pests (3)	Nelson and Rosenheim, (2006), Ingerslew and Finke, (2017), Francesena *et al*.(2019)
	Interaction with competition (1)	Stav *et al*. (2010)
	Variation of cues (2)	Ninkovic *et al*. (2013), Hermann and Thaler, (2014)
	Variation among agents, pests, cues (1)	Roberts, (2014)
	Interaction with resources (2)	Wasserberg *et al*. (2013), Silberbush *et al*. (2014)
	Interaction with plant defence (1)	Thaler *et al*. (2014)
	Variation of spatial scales (1)	Lee *et al*. (2014)
	Interaction with trap cropping, variation among agents (1)	Lee *et al*. (2011)
	Variation in plant variety (1)	Cuny *et al*. (2019)
	Ability to transmit plant pathogen (1)	Tholt *et al*. (2018)
	Indirect effects on other pest (1)	Prasad *et al*. (2018)
Individual Fitness Consequences (3)	None (1)	Matsumoto *et al*. (2003)
	Variation in agents and cues (1)	Gyuris *et al*. (2017)
	Effects of enemy on survival while infected with pathogen (1)	Ugine and Thaler, (2020)
Demographic Consequences (11)	None (2)	Nelson, (2007), Xiong *et al*. (2015)
	Interaction with temperature (1)	Bannerman *et al*. (2011)
	Variation among agents (1)	Folgarait and Gilber, (1999)
	Variation among agents and pests (1)	Weisser *et al*. (1999)
	Variation of NCE pathways (1)	Fievet *et al*. (2008)
	Interaction with plant defence (2)	Kaplan and Thaler, (2012), Kersch‐Becker and Thaler, (2015)
	Non‐target effects (1)	Fill *et al*. (2012)
	Multiple‐enemy effects (1)	Bilu and Coll, (2007)
	Effects driven by commensal species (1)	Jensen and Toft, (2020)
Plant Damage (13)	None (4)	Snyder and Wise, (2000), Maanak *et al*. (2013), Jandricic *et al*. (2016), Rendon *et al*. (2016)
	Variation among agents (2)	Hlivko and Rypstra, (2003), Hogg *et al*. (2014)
	Variation among pests and agents (1)	Rypstra and Buddle, (2012)
	Interaction with plant defence (1)	Kaplan and Thaler, (2010)
	Non‐target effects, variation among agents (1)	Walzer and Schausberger, (2009)
	In field (4)	Griffin and Thaler, (2006), Thaler and Griffin, (2008), Steffan and Snyder, (2010), Hermann and Thaler, (2018)

It can be difficult to scale up enemy‐risk effect studies from measuring pest responses to the measures of biocontrol efficacy, including effects on pest population dynamics or crop yield, as these typically require longer timescales and broader spatial scales (Hermann and Landis, 2017). However, when moving from pest–agent interactions to the harvest and sale of a crop, there are many steps where the enemy‐risk effects may attenuate (Hamburg and Hassell, 1984; Godfray and Waage, 1991; Collier and Van Steenwyk, 2004; Kaplan *et al*., 2014). Additionally, there may be many interacting effects on pests and crop yield, ranging from environmental factors to pesticide applications. Due to these complications, enemy‐risk effect studies that do not measure outcomes beyond pest responses may not fully capture the relevance of enemy‐risk effects in pest management.

Some of the most fruitful areas for further research include (1) separating NCEs and CEs to improve predictions of pest population dynamics (see Box 4), (2) considering enemy‐risk effects that include qualitative shifts, such as spatiotemporal location, and how they interact with agricultural practices in ways that differentiate them from CEs, (3) including enemy‐risk effects in assessment of agent efficacy and non‐target impacts, (4) expanding taxonomic breadth to include more parasitoids and (5) expanding scales of study to better understand the impacts on crop production. We believe ongoing empirical work would be well served by incorporating theory from the broader study of enemy‐risk effects, which would facilitate predictions about when and where risk effects may play an important role in the efficacy of pest management programs.

Box 4 **Consequences of NCEs vs. CEs for prey and predator population dynamics**

A central difference between CEs and NCEs is their consequences for natural enemy reproduction (Abram *et al*., 2019). CEs, on the one hand, generally lead to an increase in natural enemy birth rates: an immature parasitoid develops to the reproductive adult stage by attacking and killing a host, and a predator survives and reproduces by consuming prey. NCEs, on the other hand, do not result in any increase in the natural enemy’s population, and if they reduce the victim population through increased mortality or decreased fecundity, they actually shrink the resource pool available to the natural enemy. A natural enemy that induced NCEs only would eventually go extinct, as it would never be able to reproduce. It is worth noting that a generalist enemy may impose strictly NCEs on some of its prey taxa, as long as it is able to consume other species of prey or engage in omnivory. If NCEs are not explicitly accounted for, a gap between high pest mortality and low enemy reproduction may be erroneously attributed to other causes, such as poor assimilation efficiency or natural enemy mortality.

CEs and NCEs may also vary in how their overall magnitudes at the population level are influenced by predator density. A large decrease in the number of predators may lead to a large decrease in consumption of prey, but the small number of predators may still be enough to induce significant NCEs (Carpenter *et al*., 1987). The strength of NCEs can also be linked to CEs, creating potential feedbacks between the two effect pathways (Weissburg and Beauvais, 2015). Understanding the perception of risk and thresholds prey use to make decisions can help determine how NCE strength may vary with enemy population compared to CE strength (see Box 3 for a more thorough discussion of prey perception and risk management).

The inclusion of enemy‐risk effects in models has varying effects on the resulting dynamics, ranging from increased to decreased stability, the appearance of population cycles and even the reversal of predicted trophic cascades (Abrams and Matsuda, 1997; Abrams, 2008; Peckarsky *et al*., 2008; Larsen, 2012). The classic example of predator–prey dynamics involving lynx and snowshoe hares has discrepancies between observed data and CE‐only predictions, but the inclusion of enemy‐risk effects can help improve the match between prediction and observation (Hik, 1995; Boonstra *et al*., 1998). The relative contributions of NCEs and CEs to population dynamics can vary with environmental factors and the spatiotemporal scales of study, so these interactions must be accounted for if possible. Considering enemy‐risk effects in population dynamics is not simply the addition of ecological complexity for its own sake, but a way to improve predictions of population modelling.

## ENEMY‐RISK EFFECTS AND THE EVALUATION OF BIOLOGICAL CONTROL AGENTS

A primary task of biocontrol researchers is evaluating the impact of biological control agents on target and non‐target organisms. Evaluations occur during each stage of a biocontrol project, whether the program is classical, augmentative or conservation biocontrol. First, the initial step in most biocontrol programs is to describe, as quantitatively as possible, the natural enemy community associated with a target pest; for invasive species, this may involve describing food webs in both the native and invaded ranges. Second, as part of classical biological control programs, and in some cases augmentative biological control, candidate agents need to be screened for host/prey specificity to assess the risks of non‐target impacts and to identify the most promising agent(s) for mass‐rearing and release. Finally, after classical biocontrol agents are released and established, it is important to evaluate efficacy, including effects on targets and non‐targets. The methods used in each of these stages of assessment are overlapping, and different methods can be complementary (Barratt *et al*., 2010; Furlong, 2015; Macfadyen *et al*., 2015; Van Driesche, 2016; Lövei and Ferrante, 2017). However, as shown in Table 2, many methods, especially increasingly popular sequencing‐based methods, capture only consumptive effects, and either partially or completely fail to record enemy‐risk effects. Although a narrower focus on CEs is compatible with efforts to describe trophic webs, methods that reflect both CEs and enemy‐risk effects will provide better efficacy assessments and measures of non‐target effects. As noted in the literature review section, there are relatively few documented cases of enemy‐risk effects on non‐target species, but this likely reflects a failure to investigate them. The increasing prevalence of methods like immunoassays and sequencing‐based approaches to detect direct parasitism or consumption may only exacerbate this lack of documentation. Because there is no guarantee that the magnitudes of CEs are strongly correlated with the magnitudes of risk effects, both must be included in evaluation methods, if not measured separately.

**TABLE 2:** Methods used, either singly or in combination, to evaluate the impact of biological control agents on target and non‐target organisms

Method	Useful for predators, parasitoids or both	Measures consumptive effects?	Measures non‐consumptive effects?
Artificial sentinel prey models (e.g. clay caterpillars) evaluated for removal or marks of attack	Mostly Predators	✓	✗
Live tethered or outplanted sentinel prey/hosts (usually immobile stages, like eggs or pupae; but also confined larval stages)	Both	✓	✗
*Post hoc* assessment of natural enemy impact via detection of bite‐marks or other physical damage to prey	Predators	✓	✗
*Post hoc* assessment of natural enemy impact via detection of distinctive host remains, host‐feeding tubes or damage, remains of developing parasitoids (egg chorions, larval or pupal exuvia, meconia, cocoons), or distinctive parasitoid or host emergence holes	Parasitoids	✓	✗
Dissection of hosts to record parasitoid eggs, larvae or pupae; or rearing of hosts	Parasitoids	✓	✗
Monoclonal antibody‐ELISA or DNA‐based assays of hosts to detect internally developing parasitoids	Parasitoids	✓	✗
Gut content analyses – detection of prey remains using simple dissections and visual inspection	Predators	✓	✗
Monoclonal antibody‐ELISA, immunomarking or DNA‐based assays of consumer gut contents	Predators and host‐feeding parasitoids	✓	✗
Focal observations of prey/hosts, using human observers or video cameras	Both	✓	Partially*
Field life table construction by repeated sampling of a cohort of developing hosts/prey to quantify survival and rate of development from eggs to adults; often used with immobile hosts/prey	Both	✓	Partially†
Short‐term (i.e. too short for prey reproduction) mesocosm assays using hand removal or caging treatments to contrast the effects of natural enemy presence/absence; response variable = prey survival	both	✓	Partially†
Long‐term (i.e. long enough to permit substantial prey reproduction) mesocosm assays using hand removal or caging treatments to contrast the effects of natural enemy presence/absence; response variable = prey population size or growth rate	both	✓	✓
Experimental removal of natural enemy populations using selective insecticides; response variable = prey/host population size or growth rate	both	✓	✓
Experimental addition of natural enemy populations by controlling ants that otherwise exclude the nature enemy; response variable = prey/host population size or growth rate	both	✓	✓
Observational field methods comparing natural enemy present vs. absent (e.g. in classical biocontrol settings: pre‐ vs. post‐release, or release site vs. non‐release site); response variable = prey/host population size or growth rate‡	both	✓	✓

* Focal observations might reveal some NCEs related to the expression of anti‐predator behaviours, although would be unlikely to quantify the costs of such behaviours.

† This method could capture the costs of some NCEs if those costs were expressed through a reduction in developmental survival rates.

‡ Purely correlative studies examining associations between densities of predators and prey or hosts and parasitoids are also sometimes reported. But, without additional evidence of a causal link (and support for the direction of causality) such studies are often open to multiple interpretations. Thus, we omit them from the current discussion.

Projecting the non‐target impacts of a candidate classical biological control agent is quite challenging. It is difficult to canvas what is often a very broad array of possible non‐target species, each of which needs to be brought into quarantine, reared and tested for vulnerability to attack. Furthermore, it is increasingly acknowledged that not just direct impacts, but also possible indirect effects of an introduction should be assessed, including the potential for competition, IGP and apparent competition effects (Hajek *et al*., 2016; Heimpel and Mills, 2017). To this already imposing prospect, we add that it may be important to consider enemy‐risk effects. In some cases, even the simple in‐quarantine host‐range testing protocols using small and simplified microcosms and short exposures to natural enemies can reveal some evidence of enemy‐risk effects. For example, host‐range tests of candidate parasitoid species may reveal elevated mortality of individual hosts that do not produce parasitoid offspring (e.g. Abram *et al*., 2016; Bulgarella *et al*., 2017); in these cases, hosts may die following parasitoid probes without oviposition, or parasitoid oviposition may lead to early mortality of both the host and the parasitoid eggs prior to any consumption of the host. In some cases, such parasitoid‐generated host mortality has been found in host species on which parasitoids never successfully produce offspring (Hoddle and Pandey, 2014; Valente *et al*., 2017), emphasising that parasitism rates alone may not suffice to capture non‐target effects (Abram *et al*., 2019). Depending on response variables measured in target or non‐target hosts or prey, including altered movement or microhabitat selection, development rates, feeding behaviour or reproduction, other risk effects could potentially also be detected in a quarantine setting, but current host‐range testing generally sidesteps the possible importance of these effects. Like indirect effects, however, many possible enemy‐risk effects, including those expressed via longer range movements, are not readily evaluated within a quarantine facility.

More encouragingly, many widely used protocols for assessing the efficacy of biological control measure either target (or non‐target) population density as the primary response variables (Table 2), thereby capturing the combined influences of CEs and NCEs. This is particularly true in studies of conservation biocontrol, which also frequently incorporate larger spatiotemporal scales and whole communities of enemies. Although it may sometimes be of academic interest to separate the roles of CEs and NCEs (but see Box 4), these protocols accomplish the central objective of capturing the full range of pathways through which natural enemies may contribute to herbivore population suppression.

## ECO‐EVOLUTIONARY EXPERIENCE AND RESPONSES TO BIOLOGICAL CONTROL AGENTS

Recent research on prey responses to exotic enemies emphasises the importance of prey’s eco‐evolutionary experience (EEE) with enemies (Blumstein, 2006; Cox and Lima, 2006; Sih *et al*., 2010; Saul and Jeschke, 2015; Trimmer *et al*., 2017; Carthey and Blumstein, 2018; Ehlman *et al*., 2019). By ‘eco‐evolutionary experience’ we mean either an evolutionary history, or an earlier ecological (developmental) history that allowed prey to either evolve or learn to cope with a predator. Naïve prey that lack previous experience with a novel predator often respond insufficiently, suffering heavy predation (high CEs). Examples include the devastating impacts of novel predators (humans, other mammals, brown tree snakes) on naïve island prey, or of novel predatory fish on naïve prey in previously fishless lakes (Cox and Lima, 2006). For classical biological control, the expectation is that if the target pest has had an extensive evolutionary history with the imported enemy, it will likely exhibit adaptive responses (and thus NCEs) that reduce CEs. In contrast, non‐target prey that have not had previous EEE with the biocontrol agent might exhibit much weaker, if any, anti‐predator response. If the predator can attack these non‐target prey, then the biocontrol agent might prefer and exert strong CEs on non‐target prey and less consumptive impact on the targeted pest.

Some naïve prey, however, exhibit appropriate responses to novel predators. One key factor is the prey’s past history not with the specific novel predator, but with predation pressure in general. Prey that have experienced little predation pressure of any sort tend to be bolder and thus exhibit weaker response to novel predators, as compared to those that have evolved with moderate to heavy predation pressure (Ferrari *et al*., 2015). Therefore, non‐target prey should be particularly vulnerable to novel biocontrol agents if those prey species have evolved with little predation (demonstrated by novel invasive social insects in Hawaii; Wilson *et al*., 2009; Krushelnycky *et al*., 2017). Recent work adds that if prey have experienced persistent high predation risk, then they should also be bold, not cautious. If predators are persistently present, prey cannot hide indefinitely, and should only respond strongly to cues that indicate particularly high impending risk (Trimmer *et al*., 2017; Ehlman *et al*., 2019). Additionally, though we focus on arthropod pests, work on invasive plants suggests that invasive species facing no top‐down pressure may evolve to devote fewer resources to anti‐enemy responses and more to competitive ability (Blossey and Notzold, 1995). This process may be rapidly reversed upon the reintroduction of natural enemies through biological control programs, with invasive species rapidly developing anti‐enemy responses that could drastically change the initial CE–NCE ratio (Stastny and Sargent, 2017).

Another key factor in predicting prey response to an introduced biocontrol agent is its similarity to familiar, native predators. Even if non‐target prey have never experienced the particular novel predator, the ‘cue similarity’ hypothesis posits that if the introduced predator resembles familiar predators, ‘naïve’ prey are likely to respond (Sih *et al*., 2010; Saul and Jeschke, 2015). Understanding the sensory/cognitive ecology of how target versus non‐target prey perceive risk from biocontrol agents is then key (see Box 3). Even if prey correctly perceive the risk and respond, they can still suffer heavy predation if they show an inappropriate response (e.g. freeze when they should flee) or if their response is ineffective (e.g. they flee but the predator is too fast; Sih *et al*., 2010; Carthey and Blumstein, 2018). Sih *et al*. (2010) suggested that the effectiveness of naïve prey responses to novel predators should depend on the functional ‘attack mode’ similarity of novel and familiar predators, and on whether prey rely on generalised responses (that work well against a broad range of predators) or specialised ones (that work very well, but only with specific predators). If the novel predator exhibits cue similarity but attack mode dissimilarity to familiar predators, it might induce both strong but ineffective responses that result in high CEs and high NCEs. This scenario could be ideal for suppressing target prey, but disastrous if it applies to non‐target prey.

A community‐level prediction is that prey should be more likely to respond well to a novel predator if the prey have EEE with a greater diversity of predator archetypes (Blumstein, 2006; Cox and Lima, 2006; Ehlman *et al*., 2019). If prey have EEE with only one main type of predator, they might exhibit predator‐specific defences. In contrast, if either target or non‐target prey have EEE with a broad range of predators, they should be more likely to exhibit a diversity of specialised and generalised defences that could be effective against novel biocontrol agents.

Finally, it is possible that contemporary evolution could occur during a long‐term biocontrol relationship. While there are examples of evolved resistance to parasitism through enhanced immune responses (Berberet *et al*., 2003), we know of no cases where arthropod pests evolve anti‐enemy responses to biocontrol agents. Hufbauer and Roderick (2005) thoroughly reviewed microevolution in biocontrol, which may provide insights along with those gleaned from evolution of prey responses to invasive predators. Studying this directly in biocontrol systems would require measuring enemy‐risk effects over long timescales, which could become a routine part of long‐term efficacy studies.

## SPATIOTEMPORAL ASPECTS OF ENEMY‐RISK EFFECTS

Enemy‐risk effects and direct consumptive effects frequently occur on different spatiotemporal scales, with many risk effects occurring over larger areas and longer times than CEs. This means that many studies focusing on CEs lack the scale necessary to capture enemy‐risk effects, a topic that has been reviewed elsewhere (Hermann and Landis, 2017) and covered with respect to biological control in Table 2. Beyond expanding the scales of biocontrol enemy‐risk effect research in the future, current theory and evidence from the broader literature may help biocontrol practitioners conceptualise and predict how enemy risk affects pest abundance and interactions with other pest management measures in time and space.

Just as pests act within a ‘landscape of fear’ shaped by enemy cues that are heterogeneous through time and space (Laundré *et al*., 2001), agricultural landscapes exhibit spatiotemporal variability across multiple scales. Agroecosystems are spatially heterogeneous at the within‐plant, between‐plant, within‐field and between‐field scales, especially when farmers use practices such as intercropping or planting hedgerows. They also change throughout time, as many crops undergo a relatively predictable growth pattern, changing in vulnerability to various pests and in their spatial structure. Farmers apply pesticides, irrigate and harvest crops according to schedules, creating temporal patterns of disturbance. By superimposing the temporally variable landscape of risk and the temporally variable agricultural landscape, we may be able to integrate enemy‐risk effects into predictions on interactions between biocontrol agents and other IPM strategies. We outline specific ways in which enemy‐risk effects in space and time may interact with agricultural practices in the following sections.

### Enemy‐Risk Effects in Space

At smaller spatial scales, enemy risk may alter microhabitat use as pests seek refuges or move to lower quality parts of the plant (Lee *et al*., 2011; Paterson *et al*., 2013; Calvet *et al*., 2018). Pest fitness may be affected by decreased foraging time due to refuge use or consistent foraging on lower quality resources. Some pests, particularly aphids, will drop off a plant in response to enemy risk (Humphreys and Ruxton, 2019). This behaviour incurs significant costs, as dropping reduces feeding time (Nelson and Rosenheim, 2006; Nelson, 2007). It may also expose pests to a new set of mortality sources, such as ground‐dwelling enemies or increased exposure to extreme temperatures. Conversely, increased refuge use due to enemy risk may decrease pesticide exposure (Jallow and Hoy, 2005; Martini *et al*., 2012). Additionally, shifts in microhabitat use by pests may reduce the reliability of field sampling methods based on the inspections of certain parts of the plant (Southwood and Henderson, 2000).

At larger spatial scales, enemies may influence pest dispersal and habitat selection at within‐field and between‐field scales. Foraging models, such as the Ideal Free Distribution (IFD), are often used to predict pest movement and abundance within a patchy habitat, but the inclusion of mobile enemies and prey perception of enemies can drastically alter those predictions (Sih *et al*., 1998; Brown and Kotler, 2004; Fraker and Luttbeg, 2012). Natural enemies can change the threshold at which pests disperse, either increasing dispersal by making patches riskier, or decreasing dispersal by making the movement between patches riskier (Sih and Wooster, 1994; Hammill *et al*., 2015). Modelling work has shown that this can lead to seemingly counterintuitive results at the metapopulation level; if prey immigration is not affected by enemy presence, but emigration is reduced by it, then prey density can be higher in patches with enemies. Whether or not natural enemy distributions match the distributions of their prey can depend on mobility of the pests and enemies, the resource needs of each and other density‐dependent effects for each population (Winder *et al*., 2001; Nachman, 2006; Pearce and Zalucki, 2006). In general, understanding how natural enemies affect spatial patterns of pest abundance, such as higher density near field borders, may allow for more precise pest sampling and pesticide spraying, increasing the efficacy and cost effectiveness of these methods. Boxes 5, 6 and 7 all describe particular cases in which enemy‐induced dispersal aids or hinders specific pest management goals, including disease transmission, pesticide resistance and trap cropping.

Box 5 **Enemy‐risk effects and biological control of vectors of plant disease**

One of the most damaging ways that insect herbivores affect their host plants is by acting as vectors of plant pathogens. Biological control agents can clearly slow the spread of vectored pathogens by suppressing vector population densities; as both consumptive and non‐consumptive effects can depress population growth rates of insect vector populations, both can contribute to this ecosystem service (Landis and Van der Werf, 1997; Moore *et al*., 2009; Finke, 2012; Long and Finke, 2015; Clark *et al*., 2019).

However, it is now widely recognised that enemy‐risk effects may also have a somewhat counterintuitive and unhelpful influence on the epidemiology of insect‐vectored pathogens: in some cases, anti‐enemy behaviours may involve increased movement of insect vectors on both local and regional scales, accelerating disease transmission. Thus, the net effect of biological control on disease prevalence can be negative, neutral or positive, depending on the relative magnitudes of consumptive effects and enemy‐risk effects and the details of the interactions (Finke, 2012; Crowder *et al*., 2019). The empirical record has shown that outcomes can depend on the identity of the biocontrol agents, the herbivore and the pathogen (Nelson and Rosenheim, 2006; Belliure *et al*., 2011; Dumont *et al*., 2015; Clark *et al*., 2019); in particular, predator–prey interactions that result in strong prey dispersal in response to predation risk or actual predator attacks often result in short‐term increases in disease transmission any time pathogen acquisition and transmission by the vector is not interrupted by the decision to leave a feeding site.

The empirical literature shows that a widespread response of insect vectors of plant disease to predator presence and, especially actual predator attacks is to move away from the attack site via local movements (Weber *et al*., 2006; Belliure *et al*., 2011; Hodge *et al*., 2011; Dáder *et al*., 2012; Long and Finke, 2015). Aphids, which vector more than half of all plant viruses, release alarm pheromones when attacked by predators, causing clone‐mates to run away or, in some cases, to drop from the host plant (Vandermoten *et al*., 2012). Especially in cases where disease transmission requires rapid movement between two host plants (common for viruses that are transmitted via transient contamination of aphid mouthparts), this can accelerate disease transmission.

Predators can also shape longer distance movements via two potentially offsetting processes. First, many herbivores show density‐dependent induction of winged morphs or other forms of density‐dependent dispersal (Denno and Peterson, 1995; Pepi *et al*., 2016); in this case, suppression of vector population densities via consumptive or non‐consumptive effects has the potential to slow disease spread (Michaud and Belliure, 2001). Second, however, many herbivores also induce winged forms in response to detection of predator cues, including, for aphids, alarm pheromones (Weisser *et al*., 1999; Mondor *et al*., 2005; Vandermoten *et al*., 2012), potentially leading to substantial increases in potential for disease transmission over larger spatial scales. Although experimental studies have demonstrated the potential for both of these effects, how this plays out in nature is unknown.

The preponderance of evidence from experimental studies supports the hypothesis that natural enemies accelerate disease transmission in crop plant populations (Long and Finke, 2015). However, because most published studies are quite short duration, they can reveal the immediate effects of increased vector movement, but may underestimate the importance of vector population suppression, which often requires multiple generations of predator–herbivore interactions. Also, because most studies have been performed in laboratory or greenhouse settings, the importance of predators as elicitors of vector movement may be exaggerated relative to its true effect in the field, where many other factors can trigger the same trivial movements (e.g. effects of wind, mechanical disturbances and contacts with other herbivores; Bailey *et al*., 1995; Nelson and Rosenheim, 2006). Nevertheless, it is clear that biological control can be a double‐edged sword when directed against disease vectors.

Box 6 **Enemy‐risk effects, between‐plant movement and insecticide resistance management**

Predator‐induced between‐plant movement by herbivores can disrupt schemes that are intended to delay the evolution of resistance to insecticides. A significant recent change in agricultural pest management has been the introduction of crop plants genetically engineered to produce their own insecticidal proteins, derived from the bacterium *Bacillus thuringiensis* (‘*Bt*’; Tab ashnik *et al*., 2013). Although *Bt* crops can reduce the need for widespread applications of insecticides, planting a crop that constitutively produces an insecticidal toxin is a recipe for rapid evolution of resistance. To reduce this risk, evolutionary biologists working with regulators and seed companies designed and implemented the ‘high dose, refuge’ strategy of resistance management. Assuming a monogenic basis for resistance with susceptible allele *S* and resistance‐conferring allele *R*, a ‘high dose’ means that both susceptible homozygotes (genotype SS) and heterozygotes (RS) are killed on *Bt* plants. Only the rare resistant homozygotes (RR) can survive. The ‘refuge’ refers to a planted block of non‐*Bt* plants, which are expected to produce relatively large numbers of SS individuals. The rare RR homozygotes surviving on *Bt* plants are then expected to mate with one of the abundant SS individuals developing in the refuge, and the offspring (genotype RS) are subsequently killed on the *Bt* crops, removing R alleles from the population. In this way, the models suggest, resistance can be dramatically delayed (Tab ashnik *et al*., 2013).

A key problem, however, has been farmer compliance with planting the block of non‐*Bt* refuge plants (Carroll *et al*., 2012; Garcia *et al*., 2016). In response to this, seed companies have introduced the notion of a ‘refuge in a bag’: planting seed is sold as a mixture of *Bt* and non‐*Bt* seed, which generates a field with spatially interspersed *Bt* and non‐*Bt* plants. This approach is now being adopted on a global scale (Tab ashnik *et al*., 2013; Carrière *et al*., 2016). But if pests move frequently between plants in response to unsuccessful predator attacks, two problems are introduced (Mallet and Porter, 1992; Carroll *et al*., 2012; Carrière *et al*., 2016). First, the efficacy of the refuge may be eroded. The refuge in a bag idea relies on the expectation that individual non‐*Bt* plants, surrounded by *Bt* plants, can still support the development of SS individuals. If, however, SS individuals move between plants, individuals beginning their development on a non‐*Bt* refuge plant may move to a *Bt* plant and be killed (Head *et al*., 2014). Second, the efficacy of the high dose may be eroded. RS heterozygotes, which must be killed under the high‐dose strategy, can survive, favouring a rapid increase in R allele frequency, in either of two ways. First, herbivores may begin their lives on a non‐*Bt* plant, where the highly vulnerable early developmental instars can be passed safely, and then move to *Bt* plants as later instar larvae, which are often more tolerant of *Bt* toxins, allowing RS individuals to survive (e.g. Head *et al*., 2014). Second, young RS individuals who start their feeding on a *Bt* plant may be exposed to toxins, but if they move to non‐*Bt* plants before they ingest a lethal dose they may survive. Thus, enemy‐risk effects of predators that cause increases in herbivore movement, even on the very small spatial scale required to move between adjacent plants, can have major effects on the evolutionary trajectory of pest populations.

Box 7 **NCEs, trap crops and push‐pull systems**

Enemy‐induced dispersal can create large‐scale shifts in spatiotemporal pest distribution, a phenomenon that may be put to use to improve pest management programs. For example, enemies that induce stable, predictable spatiotemporal pest patterns may allow for more precisely targeted pesticide applications. Another potential route is to use enemy‐induced dispersal in tandem with trap cropping or push‐pull systems. Trap cropping is the use of highly attractive ‘trap’ plants to lure pests out of the main crop, whereas push–pull systems add a repellent ‘push’ intercrop to the ‘pull’ trap crop (Cook *et al*., 2007). Enemies may be utilised as a second ‘push’, driving pests out of the main crop and into the trap crop. This effect was studied by Lee *et al*. (2011) who demonstrated an increased level of whitefly dispersal from poinsettia into the cucumber trap crop when natural enemies were present in poinsettia. Whiteflies preferred settling in cucumber over poinsettia, but once settled in poinsettia, they did not tend to move to cucumber. Of the three natural enemies tested, only one increased whitefly dispersal into cucumber, demonstrating the importance of the specific pest and enemy pairing in this scenario.

Predictable and stable movement of pests from the main crop into the trap crop may be more likely with certain combinations of enemy, pest and plant traits. Ideally, enemies would primarily occupy the main crop, making it more dangerous than the trap crop and inducing pest dispersal into the trap crop. This could occur when enemies are habitat specialists with a strong preference for the main crop, due to plant chemical cues (Reddy, 2002), oviposition site preferences (Coll, 1996; Lundgren and Fergen, 2006) or omnivorous needs (Coll, 1996; Kopta *et al*., 2012). It could also occur if enemies are relatively immobile and can be released solely into the trap crop, which could be possible with inundative or inoculative biological control. Reduction of natural enemy dispersal has been a goal in other contexts, such as releasing wingless ladybirds to prevent them from leaving the focal field (Lommen *et al*., 2008), and it is possible that similar efforts could work at a within‐field scale as well.

Complications may arise if enemies do not primarily occupy the main crop, instead preferring the trap crop, the spaces between crops or matching pest abundance. If the enemy prefers the trap crop, it may have the opposite effect as intended, reducing pest preference for the trap crop and increasing abundance in the main crop. However, if enemies prefer the trap crop, but pests still disperse into it, the trap crop may still be effective, and enemies may then have strong effects on the pests that establish there. If enemies, perhaps ground‐dwelling predators, prefer spaces between crops, then they may increase the risk of dispersal in any direction, reducing effectiveness of the trap crop. Finally, if enemies track pest distribution, they may induce dispersal both into and out of the trap crop. This could have a range of effects, depending on the timing of dispersal, cost of dispersal and amount of trap crop. For example, if enemies track pests, forcing them to move back and forth between trap and main crops, but dispersal is very costly, the repeated dispersal may have high fitness costs for the pest. In this case, the lack of unidirectional movement into the trap crop may be more than made up for.

Just as multiple enemies may have additive, synergistic or disruptive effects on pests, so too might natural enemies and trap cropping techniques. Pest management outcomes may be optimised with a careful consideration of pest, enemy and crop combinations, necessitating more research on this topic beyond the promising existing studies.

Arthropod movement between fields is of particular interest when considering field‐scale implementation of biocontrol. Under a classical biocontrol program, where the goal is typically for an agent to disperse widely and match the pest range, enemy‐induced dispersal may not be a cause for alarm, as the enemy would be predicted to follow its prey. However, if enemy dispersal does not match pest dispersal, certain augmentative biocontrol releases may simply result in the pest problem being pushed from one farm to another. For example, flightless morphs of ladybeetles have been shown to control aphid populations more effectively due to their longer residency time in the crop (Koch, 2003). However, some ladybeetles can induce strong increases in alate production (Kaplan and Thaler, 2012) and aphid dispersal, potentially exporting the pest problem.

Finally, oviposition site selection can be strongly influenced by enemy presence. Many arthropods can detect enemies when making oviposition choices and prefer low‐risk sites (Kraus and Vonesh, 2010; Livingston *et al*., 2017), which may lead to heterogeneous patterns within or between fields. If natural enemies are in fields prior to oviposition, they may even completely deter pest establishment, referred to as biotic resistance (Gruner, 2005; Wanger *et al*., 2011). This would be more likely to occur with generalist predators, since their populations may be sustained by other species prior to the arrival of the target pest. Conservation biological control, being most focused on supporting native enemy populations, utilises biotic resistance most strongly, though any natural enemy with sufficient density prior to pest establishment may help prevent establishment.

### Enemy‐Risk Effects in Time

Temporal scaling of enemy‐risk effects is complex, since pests can respond to enemies on multiple scales, and consequences of those responses can appear at multiple scales as well. Short‐term behavioural changes by pests can lead to two main categories of outcomes: there may be a long‐term fitness consequence of short‐term changes, or there may be compensation for the short‐term effect in the long‐term. Other pest responses occur only over a longer timescale, such as changes in life‐history events. The goals of a biocontrol program affect the importance of different enemy‐risk effects across time.

Short‐term behavioural responses may lead to long‐term fitness consequences. The accumulation of small fitness losses, such as reduced feeding, mating opportunities or increased energy expenditure, can lead to long‐term reductions in population growth. Short‐term reductions in feeding rate during a vulnerable life stage may also delay development, which may lead to increased pest mortality due to high CEs (Uesugi, 2015). Furthermore, if the focus of a study is solely on short‐term effects, these long‐term changes may not be measured. Similarly, if long‐term population growth is studied without looking at short‐term mechanisms, NCEs might be missed entirely, and the change in growth rate may be attributed solely to CEs (see Hermann and Landis, 2017 for a more in depth discussion of appropriate timescales).

Pests may also compensate for short‐term enemy‐induced trait responses in the long‐term, leading to no NCEs and little impact on the pest population as a whole. If enemy risk is variable, pests that suffer losses in feeding or mating during high‐risk periods may be able to compensate during periods of low risk (Houston *et al*., 1993). Compensatory mortality can also occur in biological control systems, as when density‐dependent mortality is replaced by enemy‐induced mortality, leading to no overall difference in mortality (Cloutier and Bauduin, 1995; Suh *et al*., 2000). While this has been demonstrated in CEs, the same could occur for NCEs, where strong effects during one life stage lead to no difference in later population size.

Short‐term behavioural shifts alone may have a significant impact on biocontrol outcomes if they can be aligned with periods of crop vulnerability. Pests are often only damaging during a particular crop or pest growth stage (Hokkanen, 1991; Wiedenmann and Smith, 1997). The use of temporal asynchrony between crop and pest stages, achieved through precise timing of crop production, can exploit the narrowness of the crop vulnerability window to reduce pest impact (Letourneau and Bruggen, 2006). Similarly, if pest pressure can be reduced during that time through enemy‐induced behavioural responses, crop damage may be decreased regardless of impacts on pest population growth.

Some trait responses to natural enemies only occur in the long‐term, and as such, their consequences only appear in the long‐term as well. Pests can shift their life history in response to enemy risk, including increasing developmental rate (Thaler *et al*., 2012; Elliott *et al*., 2016; Rendon *et al*., 2016). Speeding up the development of a vulnerable life stage may reduce overall exposure to natural enemies, but incur costs later on. If shorter development means less time in a crop‐damaging life stage (e.g. less time spent as a crop‐feeding caterpillar), this may be beneficial to the crop, though it may also increase the rate of pest population growth. Different pests, even within the same order, may allocate risk avoidance behaviour to different life stages, either exhibiting oviposition site selection or juvenile enemy‐avoidance behaviour (Stav *et al*., 2000, 2010; Kiflawi *et al*., 2003; Brown and Kotler, 2004; Blaustein *et al*., 2005).

It is important to consider the goals of the biocontrol program when addressing temporal components of enemy‐risk effects. In a classical biocontrol program, where the goal is the long‐term establishment of the natural enemy, some level of CEs is necessary to sustain the enemy population, even if NCEs are initially very high. However, with an augmentative release, high enemy densities are expected to remain for only a short time. In this case, strong short‐term behavioural changes, such as temporarily reduced feeding, or short‐term behaviours that lead to long‐term fitness consequences may be enough to significantly impact the pest, though the enemy does not establish. For example, if an augmentative release of enemies leads to a large reduction in pest feeding during a week of high crop vulnerability, then long‐term impacts on pest population may be of little concern since the damaging behaviour itself was prevented.

## ENEMY‐RISK EFFECTS WITH MULTIPLE BIOCONTROL AGENTS

### Effects of multiple enemies on pests

An extensive literature has established that combinations of multiple predator species can have any of three outcomes on prey suppression: (1) additive, independent effects; (2) greater than additive, or synergistic effects; or (3) less than additive, or disruptive effects (Jonsson *et al*., 2017). Much of this literature has emphasised consumptive effects as the drivers of these outcomes; thus, synergistic effects may be generated by various forms of complementarity, including complementary use of space (e.g. consuming prey in different microhabitats) or time (e.g. consuming prey during different times of day or seasons), or differences in the host/prey stages or species attacked (Finke and Snyder, 2008; Straub and Snyder, 2008; Northfield *et al*., 2010), whereas disruptive effects may be generated by IGP or various forms of competitive interference (Vance‐Chalcraft *et al*., 2007).

Enemy‐risk effects may, however, also play important roles in shaping non‐additive effects of multiple predators (Sih *et al*., 1998). In particular, when prey defensive responses to one predator increase vulnerability to a second predator (‘risk enhancement’), the outcome is often predator facilitation and synergistic impacts on prey mortality. This is the case when pea aphids are attacked by combinations of the ladybird beetle *Coccinella septempunctata* and the carabid beetle *Harpalus pennsylvanicus*. Pea aphids drop off plants when threatened by the foliage‐foraging *C. septempunctata*, and despite adaptations for re‐grasping the plant as they fall (Meresman *et al*., 2017), some still reach the ground, where they are attacked by the strictly ground‐foraging *H. pennsylvanicus* (Losey and Denno, 1998). Similarly, strong risk enhancement is seen when *Tetranychus kanzawai* spider mites are driven out of their web refuges by specialised predatory mites *Neoseiulus womersleyi*, only to fall prey to ants that forage only outside of their webbing (Otsuki and Yano, 2014a).

Enemy‐risk effects can also contribute to predator interference. If defensive responses to one predator also confer protection against a second predator (‘risk reduction’), then total predation may be less than expected when both predators are present (Vance‐Chalcraft and Soluk, 2005). Alternatively, even when defensive responses appear to conflict, the presence of multiple predators may sometimes improve prey survival. For example, Meadows *et al*. (2017) showed that *Culex* mosquito larvae respond to a complex of mesopredators by diving towards the bottom of water bodies; however, in the presence of top predators, dragonfly larvae, which forage lower in the water column, diving responses by *Culex* are suppressed. Because the diving behaviour is costly, suppression of this response doubled the survival of larval mosquitoes to pupation. Thus, enemy‐risk effects often play key roles in shaping the emergent non‐additive impacts of multiple predators.

### Enemy‐risk effects and predator–predator interactions

Insect herbivores face the dual challenge of well‐defended host plants and natural enemies (Polis, 1999). It has become increasingly well established that predators must also forage for defended food resources (their prey) under the risk of predation. Enemy risk can stem from specialist higher order enemies (e.g. obligate hyperparasitoids); intraguild predators (competitors that also engage in uni‐ or bidirectional predation with the focal predator); or cannibalistic conspecifics (Polis, 1981; Polis *et al*., 1989; Rosenheim *et al*., 1995; Rosenheim, 1998; Schausberger, 2003; Wise, 2006). And, just as for herbivores, the impacts of higher order predators, intraguild predators and cannibals can be both consumptive and non‐consumptive (reviewed by Snyder and Ives, 2008; Frago, 2016). Although enemy‐risk effects expressed by predators reacting to other predators are generally viewed as adaptations to reduce their own risk of predation, in most cases it is difficult to separate benefits from reducing the costs of predation versus reducing the costs of competition, or even other costs of high density, such as transmission of diseases that have broad host ranges. Predation risk reduction can, however, be clearly identified as the driver when competition and disease can be ruled out, such as when a primary parasitoid abandons host patches where it detects pheromones produced by an obligate hyperparasitoid (Höller *et al*., 1994).

Natural enemies express a broad array of responses to their own predators. A common response is to move away from areas where predator risk is perceived; this may be measured experimentally as shorter patch residency times (Nakashima and Senoo, 2003; Meisner *et al*., 2011; Frago and Godfray, 2014), reduced oviposition or prey consumption (Agarwala *et al*., 2003; Magalhães *et al*., 2004; Meisner *et al*., 2011; Choh *et al*., 2015) or outright avoidance of patches where predators or predator‐associated cues are detected (Magalhães *et al*., 2004; Choh *et al*., 2015; Cotes *et al*., 2015; Seiter and Schausberger, 2015). Occasionally, parasitoids have been found to increase, rather than decrease, their oviposition activity in host patches with elevated predation risk, likely due to high patch quality even considering predator presence (e.g. Velasco‐Hernández *et al*., 2013). Other common responses include modulation of overall foraging activity (either increased or decreased; Magalhães *et al*., 2004; Bucher *et al*., 2014; Walzer *et al*., 2015; Hentley *et al*., 2016) and increased use of refuges (Venzon *et al*., 2000). Developmental effects include increased mortality, delayed (or sometimes accelerated) development, decreased (or sometimes increased) adult body size and shortened pre‐oviposition periods for adults (Walzer *et al*., 2015; Michaud *et al*., 2016). Compensatory growth has been recorded following the periods of elevated predation risk that slowed growth (Walzer *et al*., 2015). In many cases, predators respond not to reduce their own risk of predation, but rather to reduce the likelihood that their more vulnerable offspring will be attacked. Transgenerational phenotypic plasticity in response to predation risk has been recorded (Seiter and Schausberger, 2015), and in cases where predator–prey role reversals are possible, adult predators that witness a heterospecific predator attacking juvenile members of its own species may subsequently be more aggressive in reciprocal attacks on juveniles of the attacking species (Choh *et al*., 2014). Predators may even invade central locations within colonies of their prey to secure the predation risk‐reduction benefits of a selfish herd (Dumont *et al*., 2015).

What influence these responses have on the overall success of biological control is uncertain. Much of the literature is framed around the idea that anti‐enemy behaviour of intraguild prey ameliorate the impact of IGP, potentially facilitating the coexistence of multiple natural enemies, and presumably enhancing the suppression of pest populations. In the short‐term, however, anti‐predator responses that reduce potential IGP or cannibalism often results in reduced overall consumption of prey (Sih *et al*., 1998; Vance‐Chalcraft *et al*., 2007). Localised loss of contributions to biological control ascribed to non‐consumptive effects of intraguild predators or hyperparasitoids has indeed been reported (Höller *et al*., 1994; Raymond *et al*., 2000; Meisner *et al*., 2011; Frago and Godfray, 2014). But it is easier to record the potential erosion of biocontrol in a focal patch of prey than to document the possibly enhanced biocontrol elsewhere (for one study that investigated but did not find such an outcome, see Frago and Godfray, 2014). Predators that abandon patches of rich host/prey resources due to the presence of other natural enemies presumably weaken biocontrol in those patches, but may strengthen biocontrol elsewhere. Furthermore, consumptive and non‐consumptive effects have not been separated in these studies, and doing so while still assessing the overall level of biocontrol success would not be easy: treatments (e.g. mouthpart manipulations) that could be applied to an intraguild predator to eliminate CEs imposed on an intermediate predator would also, unfortunately, eliminate CEs on the shared herbivore prey. Studies of hyperparasitoids could avoid this problem. In some cases the herbivores themselves have been shown to recognise localised enemy‐free space generated by hyperparasitoids and to respond with elevated *per capita* reproductive output, perhaps as a consequence of reduced expression of costly anti‐predator defences (Van Veen *et al*. 2001). To our knowledge, no one has attempted to measure or model the global effects of fear‐mediated redistribution of natural enemies (but see Northfield *et al*. 2017 for a model that could provide a useful framework for such an investigation).

## ENEMY‐RISK EFFECTS AND BOTTOM‐UP EFFECTS

Interactions between top‐down and bottom‐up pressures have received much attention in broader natural enemy ecology, but specific breakdown of CEs and NCEs has been less common (but see Kaplan and Thaler, 2010, 2012; Thaler *et al*., 2014). A general framework for understanding the role of plant defences in altering the CE:NCE ratio focuses on the cost‐benefit ratio of engaging in anti‐enemy behaviour. An enemy‐avoidance behaviour that reduces foraging time may have a higher relative cost if food quality is low, leading to a reduction in that behaviour and resulting NCEs. The degree to which plant defences shift the trade‐off between foraging and enemy avoidance can depend on whether the pest is a generalist or specialist (Kaplan *et al*., 2014). Though a reduction in only NCEs would shift the CE:NCE ratio towards consumptive effects, plant defences can also affect the rates of enemy consumption. Generalist enemies may reduce consumption of a particular prey if plant quality or defences reduce prey biomass, prey quality or the chemical cues used by enemies to locate prey (Kersch‐Becker *et al*., 2017).

Bottom‐up effects do not always affect anti‐enemy behaviours simply by changing the cost‐benefit ratio of those behaviours. Additive effects may be possible if pests respond to plant defences and enemy risk in qualitatively different ways. For example, phytohormones have been shown to reduce aphid population growth, while natural enemies induce the production of winged morphs (Kaplan and Thaler, 2012). Here, the pathways operate independently, leading to additive effects of anti‐enemy behaviour and plant defence. In other studies, short‐distance dispersal and plant defences have been shown to interact strongly, with low plant quality and natural enemies synergistically increasing aphid dispersal (Kersch‐Becker and Thaler, 2015). Additionally, the effects of reduced plant quality and NCEs may occur on longer timescales than CEs. Pests can exploit these longer timescales by engaging in compensatory mechanisms to reduce the overall negative effects. Caterpillars facing predation risk can reduce their feeding rate but temporarily increase conversion efficiency to maintain a normal growth rate (Thaler *et al*., 2012). However, this cannot continue forever and may be dependent on the threat duration (Kaplan *et al*., 2014).

Finally, many biocontrol agents are omnivorous, meaning plant defences may affect their fitness directly. If high‐quality plants increase omnivorous enemy populations, consumption of prey may increase. However, high‐quality plants may also reduce the omnivore’s need to forage for prey, reducing *per capita* consumptive rates and NCEs. The interactions between plant defences and natural enemies are numerous, including risk effect pathways and others not discussed here, which have been more thoroughly reviewed elsewhere (Pappas *et al*., 2017). Due to these complexities, studies aiming to assess enemy‐risk effects in the field should consider what interactions with bottom‐up effects may occur.

## CONCLUSION

The study of enemy‐risk effects has advanced greatly in the past two decades, developing into a more fully realised field, incorporating theoretical frameworks, many experimental methods and even predictive models. However, the field of biological control is still catching up to broader natural enemy ecology, and the incorporation of enemy‐risk effects into the biocontrol framework is still in its infancy. There is a significant body of research documenting the importance of risk effects in biocontrol systems, but there is much room to grow beyond this. We have outlined several areas in which risk effect literature may provide insight into biocontrol practice, and hope that further studies will investigate specific interactions between enemy‐risk effects and IPM programs more thoroughly.

Community ecologists likewise can find, in biological control systems, rich examples where the consequences of risk effects play out in well‐characterised predator–prey systems, including both coevolved versus novel predator–prey associations. Agricultural systems provide ideal settings for examining both the shorter‐ and the longer term consequences of risk effects, on both smaller and larger spatial scales. Opportunities exist to examine how risk effects shape trophic cascades, the distributions of prey populations in space and even microevolutionary responses to plant defensive traits.

One of the most crucial aspects of the merging of the fields will be broadly considering biocontrol of arthropods as an inherently behavioural issue. A focus on preventing unwanted and damaging pest behaviour, whether through killing pests or changing their behaviour, broadens the scope of interactions that may be utilised in biological control. The historical focus on population density is no longer sufficient in light of research demonstrating the importance of enemy‐risk effects and how they can cascade to the level of plants.

Studies of risk effects in biocontrol systems should also include more holistic studies of the numerous interactions, either synergistic or antagonistic, between pest behaviour and broader IPM practices. Studies in this area can simultaneously investigate core ecological concepts and provide more concrete suggestions for biocontrol practitioners.

Finally, we recognise that it may not be feasible to investigate all possible enemy‐risk effects in a given agroecosystem when attempting to predict the effects of a biocontrol agent, which is why we propose the incorporation of theory and predictive models from risk effect research into biocontrol decision‐making processes. By considering the evolutionary history of the pest, bottom‐up effects of the crop and spatiotemporal dynamics of the agroecosystem, pest management programs may be able to predict the relative importance of various types of risk effects and how they may interact with management practices. Just as other detailed aspects of pest and agent biology are incorporated into management decisions, we advocate for the inclusion of enemy‐risk effect knowledge as well.

## AUTHORSHIP

All the authors discussed ideas and structure, wrote sections of the manuscript, reviewed literature and contributed substantially to revisions.

### Peer Review

The peer review history for this article is available at https://publons.com/publon/10.1111/ele.13601.

## Supporting information

Supplementary Material

## Data Availability

No original data were used in the manuscript, and all search terms and articles from the literature review are described and cited.
