# Supplementary material for: Bugs scaring bugs: enemy‐risk effects in biological control systems
Source: Ecol Lett. 2020 Sep 9;23(11):1693–714. doi: 10.1111/ele.13601 (PMC7692946; doi:10.1111/ele.13601)
Supplement: Supplementary file 1 — Supplementary Material [file ELE-23-1693-s001.docx]

**Table 1. Studies of NCEs in biological control systems**

Combinations of the search terms “biological control”, “biocontrol”, and “pest” with the terms “non-consumptive”, “nonconsumptive”, “non-lethal”, “nonlethal”, “sub-lethal”, “sublethal”, “risk effect*”, “anti-predator”, or “anti-predator.”

| **Agent** | **Pest** | **System** | **Study Location** | **Level of Study** | **Citations** |
| --- | --- | --- | --- | --- | --- |
| *Pseudacteon spp.* (Phorid) | *Solenopsis richteri* (Fire ant) | Natural | Field | Behavioral response, demographic consequences, variation among agents | (Raymond *et al.* 2000; Meisner *et al.* 2011; Frago & Godfray 2014) |
| *Phytoseiulus persimilis* & *Neoseiulus californicus* (mite) | *Tetranychus urticae* (spider mite) | Greenhouse | Greenhouse mesocosm | Behavioral response, variation among agents | (Pallini *et al.* 1999) |
| *Coccinella septumpunctata* & *Adalia bipunctata* (ladybirds) | *Acyrthosiphon pisum* (pea aphid) | Broad bean | Lab mesocosm | Morphological response, pest populations, comparison among agents, comparison among pest morphs | (Weisser *et al.* 1999) |
| *Hogna helluo*(large wolf spider), *Pardosa milvina*(smaller wolf spider), *Cyclotrachelus sodalis* (carabid) | *Diabrotica undecimpunctata howardi* (spotted cucumber beetle) | Squash | Lab mesocosm | Behavioral response, whole plant damage, | (Snyder *et al.* 2000) |
| *Pseudacteon* (Phorid) | *Solenopsis invicta* (Fire ant) | Natural | Lab arena | Behavioral response, variation among agents | (Wuellner *et al.* 2002) |
| *Gambusia affinis* (mosquitofish) | *Culex pipiens* (mosquito) |  | Outdoor pools | Behavioral response (oviposition) | (Angelon & Petranka 2002) |
| *Achaearanea tepidariorum* (tangle weaver) *Arigope trifasciata* (orb weaver) | *Popilliae japonica* (Japanese beetle),  *Epilachna varivestis* (Mexican bean beetle), *Popilliae japonica* (Japanese beetle) | Soybean | Lab arena | Plant damage, variation among agents | (Hlivko & Rypstra 2003) |
| *Aphytis yanonensis* and *Coccobius fulvus* (parasitoid wasps) | *Unaspis yanonensis*(arrowhead scale) | Citrus | Field cages and lab arenas | Behavioral response, individual fitness | (Matsumoto *et al.* 2003) |
| Various predators | *Acyrthosiphon pisum*(pea aphid) and *Aphis gossypii*(cotton aphid) | Cotton and alfalfa | Field and field arenas | Behavioral response, comparison among pests and agents | (Nelson & Rosenheim 2006) |
| *Podisus maculiventris*(stink bug) | *Manduca sexta*(tobacco hornworm) | Eastern black night- shade | Field cages | Short-term population changes, plant damage and traits | (Griffin & Thaler 2006) |
| *Aphidius colemani* (parasitoid) and *Coccinella undecimpunctata*(ladybird intraguild predator) | *Myzus persicae*  (green peach aphid) | Radish | Greenhouse mesocosm | IGP NCEs, behavioral response, pest populations, multiple enemy effects | (Bilu & Coll 2007) |
| Various predators | *Acyrthosiphon pisum* (pea aphid) | Alfalfa | Field, lab arena | Behavioral response, individual fitness, demographic consequences | (Nelson 2007) |
| *Aphidius rhopalosiphi* (parasitoid) | *Sitobion avenae* (grain aphid) | Wheat | Lab mesocosm | Behavioral response, individual fitness, demographic consequences, comparison of NCE pathways | (Fievet *et al.* 2008) |
| *Podisus maculiventris* (stink bug) | *Manduca sexta* (tobacco hornworm) | *Solanum ptychanthum* | Field cages | Behavioral response, plant damage | (Thaler & Griffin 2008) |
| *Phytoseiulus persimilis* & *Neoseiulus californicus* (mite) | *Frankliniella occidentalis* (western flower thrips) | *Phaseolus vulgaris* (French bean) | Lab arena | Behavioral response, demographic consequences, variation among agents, plant damage, non-target effects | (Walzer & Schausberger 2009) |
| *Notonecta maculata* (backswimmer) | *Culiseta longiareolata* (mosquito) | temporary pools | field mesocosm pools | Behavioral response | (Silberbush *et al.* 2010) |
| *Anax imperator* (dragonfly) | *Culiseta longiareolata* (mosquito) | desert pools | field mesocosm pools | Behavioral response, interaction with competition | (Stav *et al.* 2010) |
| *Hippodamia convergens* (lady beetle) and *Nabis alternatus* (damsel bug) | *Leptinotarsa decemlineata* (Colorado potato beetle) | potato | lab arenas, field | Behavioral response, variation among agents | (Ramirez *et al.* 2010) |
| *Podisus maculiventris* (stink bug) | *Manduca sexta*(tobacco hornworm) | Tomato | Field cages and lab arenas | Behavioral response, plant damage, plant defenses | (Kaplan & Thaler 2010) |
| *Notonecta maculata* (backswimmer) | *Anopheles gambiae* (mosquito) | Freshwater pools | Lab arena | Behavioral response | (Warburg *et al.* 2011) |
| *Notonecta maculata* (backswimmer) | *Culiseta longiareolata* (mosquito) | temporary pools | field mesocosm pools | Behavioral response | (Silberbush & Blaustein 2011) |
| *Aphidius matricariae* (parasitoid) | *Myzus persicae*(green peach aphid) | sweet pepper | Lab arena | Behavioral response, demographic consequences | (Bannerman *et al.* 2011) |
| *Amblyseius swirskii* (mite) *Delphastus catalinae* (beetle), and *Encarsia formosa* (parasitoid) | *Bemisia argentifolii* (whiteflies) | Poinsettia | Greenhouse mesocosm | Behavioral response, interaction with trap crop, variation among agents | (Lee *et al.* 2011) |
| *Azteca instabilis* (ants) | *Hypothenemus hampei* (coffee berry borer beetle) | coffee | Field, lab arena | NCE of phorids on ant agent, behavioral response, plant damage, comparison across habitats | (Pardee & Philpott 2011) |
| *Hippodamia convergens* (lady beetle) | *Macrosiphum euphorbiae* (potato aphid) | Tomato | Field cages | Behavioral and life history response, demographic consequences, plant defenses | (Kaplan & Thaler 2012) |
| *Podisus maculiventris* (stink bug) | *Manduca sexta*(tobacco hornworm) | Tomato | Field arenas | Behavioral and physiological mechanisms | (Thaler *et al.* 2012) |
| *Aphidius colemani* (parasitoid) | *Acyrthosiphon pisum* (pea aphid) | Fava bean | Greenhouse mesocosm | Non-host behavioral response, demographic consequences | (Fill *et al.* 2012) |
| *Tetragnatha elongate* (spider) and *Bombyx mori* (silkworm) | *Popillia japonica* (Japanese beetle) & *Epilachna varivestis* (Mexican bean beetle) | Snap beans | Field/lab | Plant damage, comparison among pests, comparison among agents | (Rypstra & Buddle 2012) |
| *Harmonia axyridis* (harlequin ladybird) | *Sitobion avenae* (grain aphid) & *Rhopalosiphum padi* (cherry-oat aphid) | Wheat | Lab mesocosm | Behavioral response, plant selection, comparison among pests | (Wilson & Leather 2012) |
| *Formica rufa* (red wood ant) | *Hylobius abietis* (pine weevil) | Norway spruce | Field | Plant damage | (Maanak *et al.* 2013) |
| *Coccinella septempunctata* (seven-spotted ladybird) | *Rhopalosiphum padi* (bird cherry-oat aphid) | Barley | Greenhouse mesocosm | Behavioral response, variation with enemy density/cue duration/enemy stage | (Ninkovic *et al.* 2013) |
| *Erythemis simplicicollis* Say, *Plathemis Lydia* Drury, and *Pachydiplax longipennis* Burmeister (dragonfly nymphs) | *Aedes albopictus* (mosquito) | Temporary pools | Outdoor ovitraps | Behavioral response (oviposition), interaction with food level | (Wasserberg *et al.* 2013) |
| *Neoseiulus womersleyi* and *Euseius sojaensis* (predatory mites) and *Pristomyrmex punctatus* (predatory ant) | *Tetranychus kanzawai* (spider mite) |  | Lab mesocosm | Behavioral response, variation among agents, metapopulation dynamics | (Otsuki & Yano 2014) |
| *Podisus maculiventris* (stink bug) | *Manduca sexta* (tobacco hornworm), *Leptinotarsa decemlineata* (Colorado potato beetle), *Trichoplusia ni* (cabbage looper) | Tomato | greenhouse mesocosm | Behavioral and physiological responses, interaction with plant defense | (Kaplan *et al.* 2014) |
| *Podisus maculiventris* (stink bug) | *Manduca sexta* (tobacco hornworm) | Tomato | greenhouse mesocosm | Behavioral and physiological responses, interaction with plant defense | (Thaler *et al.* 2014) |
| *Podisus maculiventris* (stink bug) | *Leptinotarsa decemlineata* (Colorado potato beetle) | Potato | Lab arena | Behavioral response, cue comparison, | (Hermann & Thaler 2014) |
| *Anisops debilis*(predatory backswimmer) | *Ochlerotatus caspius* and *Culiseta longiareolata* (mosquitoes) | Freshwater | Outdoor pools, lab arena | Behavioral response (oviposition), interaction with habitat quality (salinity) | (Silberbush *et al.* 2014) |
| *Delphastus catalinae* (ladybird) | *Bemisia argentifolii* (whiteflies) | Cucumber | Greenhouse mesocosm | Behavioral response at multiple spatial scales | (Lee *et al.* 2014) |
| *Anyphaena aperta*(nocturnal wandering spider), *Metaphidippus* spec. nr. *Manni* (jumping spider), *Theridion differens* (spider) | *Epiphyas postvittana* (light brown apple moth) | Australian tea tree and French broom | Lab mesocosm | Plant damage (branch), comparison among agents and size classes | (Hogg *et al.* 2014) |
| *Coccinella novemnotata* (nine-spotted ladybeetle) and *Coccinella septempunctata*(seven-spotted ladybeetle) | *Acyrthosiphon pisum* (pea aphid) | Fava bean | Lab mesocosm | Behavioral response, comparison between agents | (Hoki *et al.* 2014) |
| *Crocothemis erythraea* (dragonfly nymphs), *Ischnura evansi* (damselfly nymphs), *Aphanius dispar* (fish) | *Culiseta longiareolata* and *Culex quinquefasciatus* (mosquitoes) | Freshwater | Lab arena | Behavioral response, comparison among pests, agents, and cues | (Roberts 2014) |
| *Hippodamia convergens* (ladybeetle) | *Macrosiphum euphorbiae* (potato aphid) | Tomato | Field cages and lab arenas | Behavioral response, demographic consequences, interaction with plant defense | (Kersch-Becker & Thaler 2015) |
| *Harmonia axyridis* (ladybeetle) | *Helicoverpa armigera* (cotton bollworm) | Cotton | Lab arena | Physiological response, individial fitness, demographic consequences | (Xiong *et al.* 2015) |
| *Tasmanicosa leuckartii* (wolf spider) | *Helicoverpa armigera* (cotton bollworm) | Cotton | Lab arena, greenhouse mesocsm | Behavioral response, individual fitness, plant damage (living) | (Rendon *et al.* 2016) |
| *Neoseiulus cucumeris* (predatory mite) | *Frankliniella occidentalis* (western flower thrips) | Red kidney bean | Lab arena | Behavioral response, individual fitness, plant damage (leaf) | (Jandricic *et al.* 2016) |
| *Phytoseiulus persimilis* (mite) *& Orius majusculus* | *Tetranychus urticae* (spider mite) | Strawberry and beans | Lab arena | Behavioral and life history response, comparison among agents and plants | (Jacobsen *et al.* 2016) |
| *Aphidius colemani* and *Aphidius ervi* (parasitoids) | *Acyrthosiphon pisum* (pea aphid) and *Myzus persicae* (green peach aphid) | Fava bean/collards | Greenhouse mesocosm | Behavioral response, comparison among pests and agents | (Ingerslew & Finke 2017) |
| *Phytoseiulus persimilis* & *Amblyseius swirskii* (mites) | *Tetranychus urticae* (spider mite) | Beans | Lab arena | Behavioral response, individual fitness, comparison among agents and cues | (Gyuris *et al.* 2017) |
| *Podisus maculiventris* (soldier bug) | *Leptinotarsa decemlineata* (Colorado potato beetle) | Potatoes | Field arenas and greenhouse mesocosms | Behavioral response, individual fitness, plant damage (living) | (Hermann & Thaler 2018) |
| *Stenocorse bruchivora* (parasitoid) | *Zabrotes subfasciatus* (seed beetle) | Lima bean | Laboratory mesocosms | Physiological response, variation in plant variety | (Cuny *et al.* 2019) |
| *Aphidius ervi* (parasitoid) | *Aulacorthum solani* (Foxglove aphid) | Sweet pepper | Greenhouse | Behavioral response | (La-Spina *et al.* 2019) |
| Various spider species | *Sphenophorus spp* (billbugs) | Turfgrass | Laboratory assay | Behavioral response | (Dupuy & Ramirez 2019) |
| *Podisus maculiventris* (spined soldier bug) | *Leptinotarsa decemlineata* (Colorado potato beetle) | Potatoes | Laboratory assay | Demographic response, effect of predator chemicals on prey susceptibility to fungal infection | (Ugine & Thaler 2020) |
| *Cycloneda sanguinea, Eriopis connexa and Coleomegilla quadrifasciata* (coccinellids), *Orius insidiosus* (minute pirate bug) | *Chaetosiphon fragaefolii* (strawberry aphid), *Aphis gossypii* (cotton aphid) | Strawberry | Laboratory assay | Behavioral response, variation in predators and prey | (Francesena *et al.* 2019) |
| *Schizocosa ocreata* (brush-legged wolf spider) | *Ixodes scapularis* (blacklegged tick) | Forests | Field microcosm | Behavioral response | (Fischhoff *et al.* 2018) |
| *Tibellus oblongus* (spider) | *Psammotettix alienus* (leafhopper) | Barley | Laboratory microcosm | Behavioral response, ability to transmit plant pathogen | (Tholt *et al.* 2018) |
| *Celithemis eponina* (Halloween pennant dragonfly), *Enallagma* spp. (bluet damselfly), *Procambarus* (crayfish) | Various mosquito and midge species | Rock pools | Outdoor aquatic mesocosms | Behavioral response, variation in predators | (Staats *et al.* 2016) |
| *Phytoseiulus longipes* and *Phytoseiulus macropilis* (mites) | *Tetranychus evansi* (spider mite) | Tomato | Laboratory assay | Behavioral response, variation in predators | (Dias *et al.* 2016) |
| *Harmonia axyridis* (Asian ladybeetle) and *Bembidion lampros* (carabid beetle) | *Myzus persicae* (green peach aphid) and *Delia* spp. (root maggot) | Radish | Laboratory microcosm | Behavioral response, indirect effect on other pest | (Prasad *et al.* 2018) |
| *Drosophila melanogaster* (vinegar fly) | *Rhopalosiphum padi* (bird cherry-oat aphid) | Wheat | Laboratory microcosm | Demographic response, commensal “enemy” | (Jensen & Toft 2020) |
| Various enemies | *Plutella xylostella* (diamondback moth) | *Brassica oleracea* | Field mesocosm | Plant biomass, behavioral response | (Steffan & Snyder 2010) |
|  |  |  |  |  |  |
